# Influence of the Feedstock on the Process Parameters, Product Composition and Pilot-Scale Cracking of Plastics

**DOI:** 10.3390/ma14113094

**Published:** 2021-06-04

**Authors:** Daria Frączak, Grażyna Fabiś, Beata Orlińska

**Affiliations:** 1 Clariter Poland Sp. z o.o., 59A Żelazna Str., 00-848 Warszawa, Poland; info@clariter.com; 2 Department of Organic Chemical Technology and Petrochemistry, Silesian University of Technology, 4B Krzywoustego Str., 44-100 Gliwice, Poland; beata.orlinska@polsl.pl

**Keywords:** plastic waste, polyolefins, thermal cracking, pyrolysis, chemical recycling

## Abstract

Chemical recycling of polymers can lead to many different products and play a significant role in the circular economy through the use of plastic waste as a feedstock in the production of valuable materials. The polyolefins: polyethylene (PE) and polypropylene (PP), together with polystyrene (PS), can be chemically recycled by the thermal cracking (pyrolysis) process. In this study, continuous cracking of polyolefins and polystyrene in different proportions and with the addition of other polymers, like polyethylene terephthalate (PET) and polyvinyl chloride (PVC), was investigated at the pilot scale in terms of the process parameters and product yields. Gas chromatography with mass spectrometry (GC-MS) was used for the detailed analysis of the products’ compositions. The boiling temperature distribution and the bromine number were used for additional characterization of products. It was found that an increase of PP share caused a decrease in the process temperature, an increase of the product yield and a shift of the boiling range towards lighter products, increasing the content levels for unsaturates and branched hydrocarbons. It was observed that the addition of 5% PS, PET and PVC reduced the overall product yield, resulting in the creation of a lower-boiling product and increasing the conversion of polyethylene. An addition of 10% polystyrene increased the PP conversion and resulted in a higher product yield, without significant change in the boiling temperatures distribution.

## 1. Introduction

Plastic plays a significant role in society today. In 2018 there were almost 360 million tons of plastics produced worldwide. This was almost 10 million tons more than the previous year. Out of that volume, around 62 million tons of plastics were produced in Europe. Currently, in Europe, only about 32% of collected post-consumer plastic waste is mechanically recycled and over 40% is still incinerated [1]. The reasons include the technical limitations of mechanical recycling as well as the limited use of recyclates in some applications, like food-contact packaging. During re-processing of plastic waste, partial degradation of polymer chains takes place and mechanical properties of the plastic are changed. During extrusion, which is the most commonly used method for mechanical recycling, thermo-oxidative and shear-induced chain scission, chain branching or crosslinking of the material can take place due to the thermal conduction and viscous shearing. The tensile strength and elongation at break or impact strength are examples of parameters that can be changed. Furthermore, polyethylene and polypropylene blends face the issue of poor adhesion between polymer phases, so recycled products have mechanical properties like the Young’s moduli and elasticity reduced. Pigments, printing inks, plastic or paper labels, lubricants and addition of other unwanted polymers are the major contaminants that may additionally reduce the quality of the recyclate and cause the material to fail to meet the standards for primary application, such as food-grade safety standards [2]. That is why recyclates are usually used in lower-grade applications—for example, food packaging waste is transformed into pipes or agriculture foils. This process is sometimes called “downcycling”. Chemical recycling of polymers, defined as “conversion to monomer or production of new raw materials by changing the chemical structure of plastics waste through cracking, gasification or depolymerization, excluding energy recovery and incineration”, can play a significant role in the reduction of plastic waste volume and in enabling a circular economy through the production of valuable materials for the industry, like monomers for the production of new virgin polymers, intermediates for chemical processes and chemicals for the formulation of final goods like lubricants, degreasers or impregnation waxes [3,4]. Nevertheless, mechanical recycling should always be the prioritized solution as the process consumes less energy and chemical recycling should treat materials that are not acceptable for it. Examples of such materials include low-grade plastics, mixed polymer streams, printed flexible packaging and multilayer packaging.

Over 55% of the European demand for polymers is for polyolefins (polyethylene (PE) and polypropylene (PP)) and polystyrene (PS) [1]. Polyethylene and polypropylene can be chemically recycled directly to monomers only with limited yields [5,6]. Depolymerization of polystyrene gives higher yields of styrene monomer, but the presence of other products can cause a lowering of the average molecular weight of the polymer obtained by the polymerization of such a mixture [7]. An interesting alternative is pyrolysis (cracking) of polyolefins and polystyrene into an intermediate for the petrochemical industry, namely pyrolysis oil, which can be further fractionated into fuel components (not considered as recycling) or aromatic hydrocarbons, or upgraded to feedstock for steam cracking or final products like lubricants, solvents, oils or waxes [8,9,10,11,12,13,14,15,16,17,18,19].

During cracking of polyolefins and polystyrene, three products are received: a gas fraction, a condensable fraction (pyrolysis oil) and a residue (coke/char), in different ranges [20]. The condensable fraction may be fully liquid at ambient temperature but may also consist of wax. Pyrolysis oil’s composition also strongly depends on process conditions, like the type of reactor, the temperature and pressure of the process, the residence time and the presence and type of catalyst [21,22,23,24]. In general, the quantities of the gas product and char increase with longer residence times together with increases in the aromatics content and the paraffin-to-olefin ratio [22,23,25,26]. Longer-chain hydrocarbons content increases with the temperature of the process up to a certain point (about 500–550 °C) and then starts decreasing due to secondary reactions [27]. Ahmad et al. observed that during the cracking of PP the yield of the liquid increased from 57.27% at 250 °C to 69.82% at 300 °C along with the temperature, but further increase of the temperature caused a decrease of the liquid yields to 67.74% at 350 °C and 63.23% at 400 °C due to increased coke and gas formation. In the case of high-density polyethylene (HDPE), the yield of the liquid product has been found to be highest at 350 °C (80.88%), while at lower temperatures, solid residue yield was higher and, at higher temperatures, the gas product yield increased. [28] The presence of catalysts generally lowers the required temperature and time of reaction but also leads to higher aromatics content [23,25,29,30]. Composition of the feedstock is also an important factor. Different polymers can interact during co-pyrolysis. For example, synergistic interaction of PP and PE has been observed, resulting in higher yields or lower temperatures of cracking [21,31]. The addition of PS to PE can enhance the rate of reaction. Similar behaviour may be observed during pyrolysis of plastics with an addition of polyvinyl chloride (PVC) or complex mixtures of polyethylene, polypropylene, polystyrene and polyethylene terephthalate (PET) [23].

Currently, pyrolysis technologies exist at various scales of operation, including in commercial operations. Nevertheless, much research is still conducted using different types of reactors and process operations [32,33]. The batch and semi-batch reactors are the most popular reactors at laboratory-scale testing, with thermogravimetric analysers being an example. These types of reactors are difficult to scale up, which is why other types of reactors are being developed [34,35].

Miskolczi et al. (2009) conducted pyrolysis of waste plastics in a pilot-scale reactor. Polymers were cracked in a tube reactor at 520 °C with an hourly feed rate of 9.0 kg. An addition of 5% ZSM-5 catalyst was also tested. The use of the catalyst caused an increase of the gaseous and light fractions (gasoline and light oil) and a decrease of the heavy oil content. The gasoline and light oil fractions obtained during catalytic cracking of both HDPE and PP had less n-paraffins and significantly less vinyl olefins than the same fractions obtained during thermal cracking. On the other hand, iso-paraffin, vinylene olefin and aromatic contents were higher [36].

Miandad et al. (2016) investigated the influence of temperature and reaction time on polystyrene cracking in a pilot-scale batch pyrolysis reactor. The reactor had a capacity of 20 L and 1 kg samples were used for each experiment. In total, 76.0–78.7% of the liquid oil was produced at temperatures of 400–500 °C. Reaction time did not significantly influence the oil yield (79.0–80.7% for 60–120 min), but decreases of char and increases of gas yields were observed with the time. At 400 °C, 39% styrene, 28% ethylbenzene and 28% toluene were obtained. The composition changed at 450 °C, resulting in a higher yield of styrene (48%) and lower yields of ethylbenzene (21%) and toluene (28%). The composition of the oil produced at 500 °C was the same. Shorter and longer residence times of 75 min resulted in lower yields of styrene [37].

Park et al. (2019) conducted continuous two-stage pyrolysis of waste polyethylene. The first stage, an auger reactor, was operated at temperatures of 30 to 300 °C. The product from the first reactor was then fed into a fluidized bed reactor operating at 653 to 736 °C. A total of 22.63–35.92% of the pyrolysis oil was obtained, consisting of 80–90% aromatic hydrocarbons [38].

The objective of this research was to investigate the influence of the feedstock on process parameters, yields and product composition in a continuous, pilot-scale pyrolysis process. Based on the composition, potential applications and further processing steps were proposed. The novelties of the current work are the use of a continuously operating reactor at a pilot scale for the thermal cracking process, the way that process parameters were chosen (stable product rate: 2 kg/h for each type of the raw material) and the presentation of the very detailed composition of products, considering identification challenges.

## 2. Materials and Methods

### 2.1. Raw Materials

Virgin LDPE (low-density polyethylene) and HDPE produced by Sabic in Geleen, The Netherlands and Riyadh, Saudi Arabia respectively, and homopolymer PP produced by Basell Orlen Polyolefins in Płock, Poland, were used for testing. A 50/50 mixture of LDPE and HDPE was used as the PE raw material. The PS, PET and PVC used were commercially available regranulates from mechanical recycling. As all materials were already in the form of 5 mm granules, there was no need for any additional pre-treatment. Samples of polymer mixtures were prepared as presented in Table 1.

### 2.2. Pilot Scale Cracking Set-Up Description

**Figure 1:**
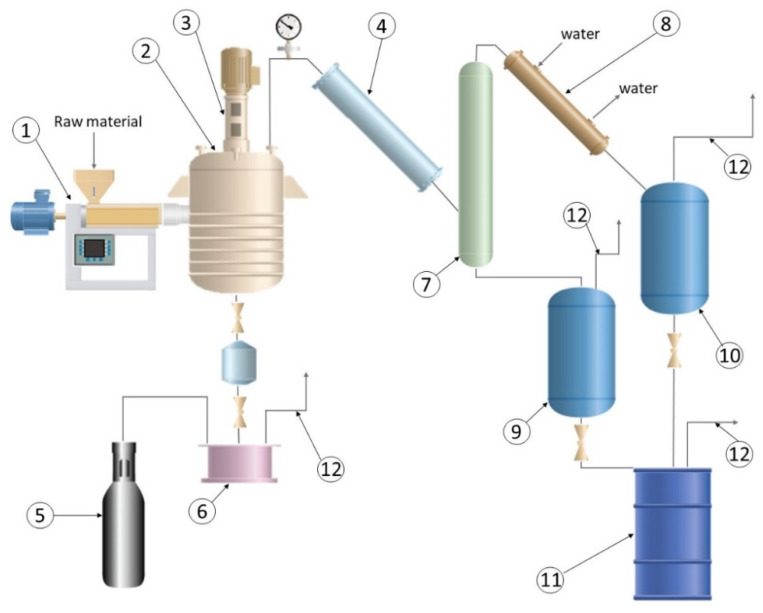
Thermal cracking unit: (**1**) extruder, (**2**) thermal cracking reactor, (**3**) stirrer, (**4**) air cooler, (**5**) nitrogen container, (**6**) residue tank, (**7**) separation column, (**8**) water cooler, (**9**) heavy fraction receiver, (**10**) light fraction receiver, (**11**) product collection, (**12**) off-gas.

Continuous thermal cracking of polymers was carried out for about 8 h in a pilot-scale set-up, presented in Figure 1, based on the modified unit invented by Podeszfa et al. [39]. Plastic raw material was fed to the extruder (1) where it was melted and heated up to 330 °C. Then, the melt was continuously introduced to a 50 dm^3^ tank reactor with stirrer (2) where cracking took place. The reactor was heated by controlled electrical heaters. Vapours of products were continuously carried over through the air cooler (4) to the separation column (7). The heavy fraction was collected from the bottom of the column in the heavy fraction receiver (9). The light fraction was cooled in the cold water condenser (8) and collected in the light fraction receiver (10). Both fractions were mixed together in a weighted product barrel (11). The process temperature was dependant on polymer type and chosen to maintain a stable production rate of 2 kg/h of the product. Residue from the cracking process was drained from the reactor in portions through a special draining system to the residue receiver (6). Gaseous products were transferred out of the unit through dedicated lines (12). The quantity of gaseous product and residue was calculated as the difference between the input weight and product weight. The whole system was purged with nitrogen from a nitrogen cylinder (5) before the process and before every drain of the residue. The cracking reactor was not purged with nitrogen during the process.

### 2.3. Cracking Product Characterization

Detailed analysis of the cracking product was conducted by gas chromatography with mass spectrometry using a Perkin Elmer Clarus 600 gas chromatograph (Shelton, CT, USA) equipped with a 30 m long and 0.25 mm diameter capillary chromatographic column and an Elite 5 MS low-polarity film of 0.5 µm thickness, along with a Clarus 600 MS spectrometer with quadrupole mass analyzer and photomultiplier. The chromatograph injector temperature of 350 °C was retained. The GC oven was programmed to hold at 40 °C for 10 min, then ramp to 320 °C at 6 °C/min and hold for 10 min. Helium was used as a carrier gas with a constant flow rate of 0.8 mL/min. A 1.0 µL sample injection and a split of 50 were used. Liquid samples were injected directly; solid and semi-solid samples were injected as 1% wt. solutions in carbon disulphide (CS2). The mass spectrometer electron energy was 70 eV, and the ion source and transfer line temperature were 200 °C. The detector was turned off in the time range of 0–2.4 min to allow the solvent to leave the spectrometer. The built in TurboMass v. 6.1.2 software from Perkin Elmer was used for data collection. Automatically integrated peaks with a calculated minimum concentration of 0.1% wt. were analyzed and counted. Chromatographic peaks were identified by means of the NIST mass spectral data library. When detailed identification was not possible due to the low probability of the proposed species, additional evaluation by characteristic ion was considered. Characteristic ions for specific hydrocarbon groups are listed in ASTM D2425 and in several studies [40,41,42,43]. The concentration of each component was calculated by dividing the proper peak height or area by the sum of all peak heights or areas, respectively, and multiplying by 100%.

The boiling range of each sample was analysed by simulated distillation (SIMDIS) following ASTM D7500 [44]. A Perkin Elmer Clarus 580 (Shelton, CT, USA), equipped with a 5 m long capillary chromatographic column with an internal diameter of 0.53 mm and a 0.1 µm thick, non-polar Col C stationary phase, in combination with an flame ionization detector (FID), was used. The temperature of the on-column injector was ramped from 40 °C to 430 °C at 20 °C/min. The final temperature was held for 9.5 min. The temperature of the oven was ramped from 35 °C to 430 °C at 15 °C/min, and the final temperature was held for 2.67 min. The detector temperature was 450 °C. Then, 1% wt. solutions of samples in carbon disulphide were injected in quantities of 0.4 µL. Helium was used as a carrier gas with a constant flow rate of 14 mL/min. For the FID, synthetic air with a flow rate of 450 mL/min and hydrogen with a flow rate of 45 mL/min were used. The built in TotalChrom v. 6.3.2 and Dragon v. 1.2.0 software from Perkin Elmer were used to collect the data and automatically calculate the boiling range.

The bromine number was analysed according to ASTM D1159 [45]. The weighted sample (around 4 g) was dissolved in the solvent. The solvent was created by mixing 714 mL of glacial acetic acid, 134 mL of dichloromethane, 134 mL of methanol and 18 mL of sulphuric acid solution (1 volume of concentrated sulphuric acid was mixed with 5 volumes of distilled water). The sample solution was then cooled down and maintained at 0–5 °C, then titrated with 0.25 M standard bromine-bromate solution. The endpoint was indicated by a sudden change in potential on an electrometric apparatus, after which the addition of 0.2 mL of bromine-bromate solution did not cause an increase of more than 5–10 mV.

## 3. Results and Discussion

### 3.1. Influence of the Raw Material Composition on the Cracking Process and Yields

Process temperatures varied in the range of 330 to 403 °C. Product and loss (residue and gas) yields were estimated and varied from 48.1 to 91.3 and 51.9 to 8.7, respectively, in percentage weight (Table 2).

In the first part of the research, the influences of different ratios between polyethylene and polypropylene were evaluated. Ratios of 100% PE and 100% PP were used as a baseline. To keep a stable product rate, a difference of over 30 °C in the process temperature was needed. This difference is required due to the difference in the polymer chain structures. In polypropylene, every monomer consists of a tertiary carbon atom, which creates more stable radicals and requires lower energy to break the bond between carbon atoms [46]. A total of 78.1% of condensable product was obtained from PE, which was lower than observed in other research (up to 93.1%) [22]. The condensable yield of the product observed for PP was higher than for PE and higher than described in the literature on other types of reactors (48.8–69.82%) [22]. The process temperatures for mixtures of PE and PP were in between the cracking temperature of PE and that of PP. It was observed that an increase of PP share caused a decrease of process temperature and losses yield, thus increasing product yield. This synergetic behaviour was in line with the findings of Dubdub and Al-Yaari for a batch process conducted in a thermogravimetric analyser (TGA) [47]. Surprisingly, the product yield in the process with Sample 5 was higher than for pure PP. This might have been caused by the higher temperature of the process with the mixture. Cracking of polystyrene was conducted at the lowest temperature, 330 °C, but also with the lowest yield—lower than described in the literature (97%) [22]. PS is known for having the lowest cracking temperature compared to PE and PP [46]. The mixture of PE, PP and PS was cracked at the same temperature as the PE/PP 50/50 mix but with a product yield 1.3% higher. The synergistic behaviour of PS with polyolefins, causing higher conversions, has been described in the literature [23,48].

The cracking process of Sample 8 was carried out next. Four hours after the start-up and once a temperature of 400 °C had been reached, the air cooler was chopped by solid material that was poorly soluble in organic solvent. Although some product was collected, a yield of only about 52% was obtained. Osman et al. have reported that, depending on process conditions (like heat rate), PET pyrolysis can already start below 350 °C [49]. PET pyrolysis can result in many different products, including terephthalic acid, which is suspected to be a reason for piping clogging [50,51]. This is why, in the next process, a smaller quantity of PET was added. Thermal cracking of the mixture of Sample 9 was conducted without any issues at 384 °C, giving 85% of the product. In the last process, a 50/50 PE/PP mixture was used as a basis to verify the influence of the addition of other plastics. Although the process temperature was only 1 °C lower, the overall product yield was lower. The results were contrary to those of Sing and Ruj (2016), who demonstrated the synergistic effect of mixtures of plastics through the fact that degradation of mixed plastics started at a lower temperature (310 °C) than degradation of individual plastics (350 °C) [52].

### 3.2. Cracking Product Characterization

**Figure 2:**
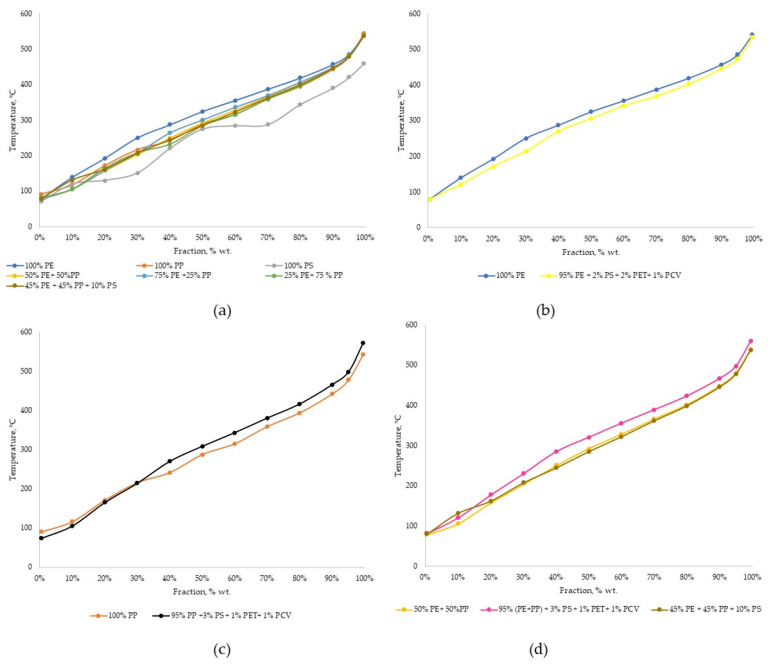
Boiling range distribution: (**a**) comparison of all samples obtained by cracking of polyethylene, polypropylene, polystyrene and their mixtures; (**b**) comparison of samples obtained by cracking of polyethylene and polyethylene with the addition of other polymers; (**c**) comparison of samples obtained by cracking of polypropylene and polypropylene with the addition of other polymers; (**d**) comparison of samples obtained by cracking of polyethylene and polypropylene 50/50 mixture and the same mixture with addition of other polymers.

The boiling range distribution for condensed products varied according to the different temperature rates (Table 3 and Figure 2).

Although the initial boiling point (IBP) and final boiling point (FBP) of Sample 1 were lower those than of Sample 2, fractions of 10% to 95% indicate that the product of polyethylene cracking was heavier than the product of polypropylene cracking. It can be concluded that the presence of PP in the feedstock not only reduces the process temperature but also causes a shift of the boiling range towards lighter products. Polystyrene cracked into the lowest-boiling product. A 10% addition of PS to the mixture of polyethylene and polypropylene did not affect the boiling point distribution significantly—it was observed that at 10% and 20%, the boiling temperature was higher for Sample 7 than for Sample 4. The addition of 5% of other plastics (PS, PET and PVC) to PP resulted in a higher-boiling product than the cracking product of pure PP. With regard to PE and the mixture of PE and PP, the addition of these plastics caused the creation of a lower-boiling product than the same polymers without additions, with similar temperatures for the cracking process.

**Figure 3:**
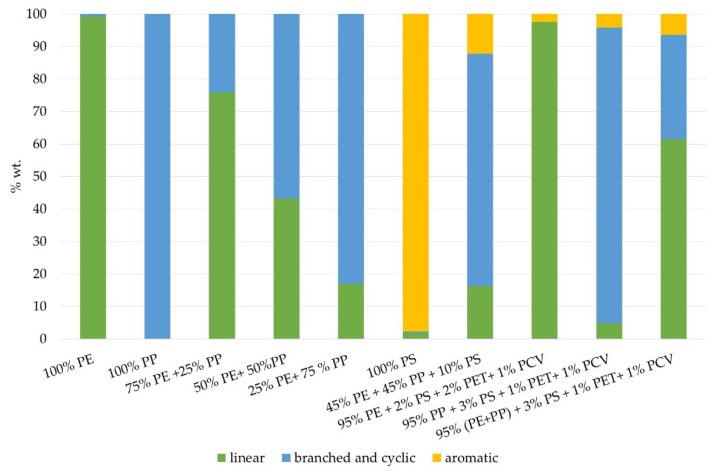
Ratios between different types of molecules.

Group compositions based on GC-MS analysis and bromine numbers of products ranged from 34.20 to 60.16 gBr_2_/100 g (Table 4). The ratio between linear, branched and cyclic, and aromatic components showed that linear component content was directly correlated to PE content in the feedstock and varied from 99% for Sample 1 to 0% for Sample 2. Aromatic content was directly correlated to PS content in the feedstock and, in samples obtained from polyolefins, no aromatic hydrocarbons were identified (Figure 3). Detailed information on the compositions is presented in the Supplementary Materials.

During the detailed analysis of the composition of the products, identification of some of the components was challenging. A chromatogram of the PP cracking product consisted of around 80 peaks with concentrations of more than 0.1%. A large majority had fragmentation ions characteristic of unsaturated and/or cycloalkanes—mainly ions 55, 69, 83 (*m*/*z*) [40,41,42,43]. The unsaturated hydrocarbons and cycloalkanes were fragmented, giving the same characteristic mass fragments. Mass spectra of these molecules (cyclo-alkanes, alkenes) differ in the intensity ratio of each fragment ion. This enables their specification and identification. However, in the case of pyrolysis products of PP, the mixture should have consisted mainly of iso-olefins. This is a result of the branched structure of polymer, which is broken into branched, unsaturated molecules. Nevertheless, some cyclic components were identified within the lightest hydrocarbons (up to 1,4,5-trimethylcyclohexane). It should be noted that each iso-olefin molecule had a few stereoisomers that were difficult to separate based on the boiling temperature because these molecules were almost identical and were eluted from the column as one peak. The mass spectra of such a mix were “disrupted” and difficult to compare to the spectra of “pure” compounds recorded in the database. The number of possible stereoisomers increased together with an increase in the molecular weight of the component. The resolution of the GC-MS system used for analysis enabled simple identification of iso-olefins with a carbon number up to C10. All the other components were added to this group based on the mass spectra analysis rather than the NIST database. For that reason, cyclo-paraffins and iso-olefins were presented together—for higher molecular weights, differentiation of these types of hydrocarbons was impossible.

A significant difference in composition was observed for products from pure polyethylene, pure polypropylene and pure polystyrene. The PE cracking product consisted of 99% linear hydrocarbons and the PP cracking product consisted only of branched and cyclic compounds, while the PS cracking product was composed of almost 98% aromatic hydrocarbons. In both the PE and PP cracking products, aromatics were not identified. Similar products have been described in other non-catalytic pyrolysis processes [10,24,26]. The PE cracking product consisted mainly of saturated hydrocarbons (over 70%) and was characterized by a bromine number of 34.20 gBr_2_/100 g. Cracking of PP led to a higher rate of non-saturates which was presented by a very high bromine number: 72.80 gBr_2_/100 g. Conversely, Ahmad et al. (2015), for example, reported a higher alkenes concentration for the product of HDPE cracking in a microreactor compared to that for PP cracking: 25.7 and 31.9%, respectively [28].

**Figure 4:**
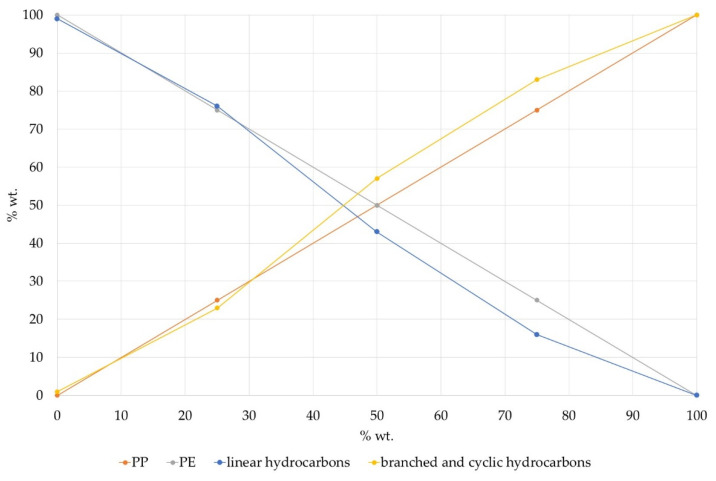
Correlation between PP content in the feedstock and hydrocarbon types in the cracking product.

It can be observed that, with an increasing polypropylene percentage in the raw material, the n-paraffin and n-olefin contents in the cracking product decrease, and the bromine number together with the iso-paraffin and iso-olefins/cycloparaffin contents increase. Although the concentration of linear and branched/cyclic components for Sample 3 was very similar, 76% and 23%, respectively, for other PE/PP mixtures the correlation between the composition of the feedstock and the types of hydrocarbons in the product was not as direct. It can be observed that, with the rise of the PP rate in the feedstock, the concentration of branched and cyclic components rose, albeit not proportionally (Figure 4). A similar non-proportional correlation between the PE rate and the linear components concentration was observed. This can be explained by the fact that PE and PP crack with different product yields even at optimum temperatures. The temperature of each process when the mixture of PE and PP was used was between the temperatures required for pure PE and for the cracking of PP. The conversion of PE was even lower. However, this method of post-cracking analysis of the PE/PP ratio in the raw material could be used for general estimation of feedstock composition. This might be important in a situation in which waste plastic with an unknown composition is used in a process, for example, in the case of a mixed plastic stream from municipal solid waste (MSW). This feedstock quality control procedure is implemented in the company Clariter to ensure the proper composition of the feedstock (within the specifications) and the optimization of the next step of the process—hydrotreatment.

Detailed analysis of the cracking products’ compositions showed that, in the case of Sample 3, none of the branched or cyclic hydrocarbons were present in the components with more than 19 carbon atoms in the chain. In Samples 4 and 5, iso-olefins/cycloparaffins were identified in heavier fractions (with higher retention times). In all samples where PE was present in the feedstock, n-paraffin was preceded by n-olefin with chain lengths of up to 23 carbon atoms. A high peak for 2,4-dimethylheptene was characteristic of samples obtained by cracking of PP and its mixtures.

The main product of PS cracking was styrene, with a yield of almost 34%. Two components with two phenylic rings connected with a hydrocarbon bridge were identified with concentrations of over 18% and 12%. All the other components were identified in concentrations lower than 10%. The presence of characteristic 2,4-dimethylheptene and n-paraffins with low concentrations indicated the contamination of the product with products from previous processes with polyethylene and polypropylene mixtures. The last eluted component was an aromatic hydrocarbon with four rings. Achilias et al. (2007) also reported that styrene was the major product of thermal cracking of polystyrene, followed by diphenyl hydrocarbon, but the concentration of monomer they identified was much higher at 63.9%. Structures with three aromatic rings were also identified [7].

An addition of 10% polystyrene to the feedstock resulted in 12.2% aromatic hydrocarbons in the product, but it should be noted that the peak from toluene (retention time (RT): 4.784 min) was combined with the peak from 4-methylheptane (RT: 4.819 min). Considering the 2.91% concentration of 4-methylheptane in Sample 4, it can be concluded that the aromatics content in the product was similar to the polystyrene content in the feedstock. Furthermore, the peak for 1,1′-(2-butene-1,4-diyl)bis-benzene overlapped with iso-olefin/cycloparaffin components, but its concentration was difficult to predict. On the other hand, only 16% of the linear components were identified, which indicated a low PE conversion; furthermore, the process temperatures, product yields and boiling temperature distributions of tests 4 and 7 were similar. Heptylbenzene was an aromatic hydrocarbon that was identified in the product but was not found in the PS cracking product. Only one polycyclic aromatic hydrocarbon was identified, with two phenylic rings. No significant difference for the bromine number was observed when compared with Sample 4, despite the 3.12% styrene and 0.89% α-methylstyrene found in Sample 7. This can be explained by the low n-olefin content—almost half of the n-olefin content found in Sample 4.

The composition of Sample 8 was very similar to that of Sample 1, despite the presence of 2.44% aromatics. Apart from the presence of aromatics, almost 5% of the linear components differed between Sample 9 and Sample 2. The biggest difference between the cracking products of polyolefins and those of polyolefins with the addition of other plastics was observed between Sample 10 and Sample 3. In Sample 10, a much lower content of branched and cyclic hydrocarbons was observed compared to Sample 3. Almost 20% more linear components were identified in Sample 10 than in Sample 3, despite the same process parameters and similar product yields. The composition of Sample 10 was also significantly different from Sample 7. A significantly higher conversion of polyethylene was observed. For both Samples 9 and 10, in which PP was present in the feedstock, the toluene peak overlapped with the peak for 4-methylheptane, increasing the aromatic content by about 2%. Furthermore, the peak for 1,1′-(2-butene-1,4-diyl)bis-benzene overlapped with the peak of an iso-olefin/cycloparaffin component that difficult to identify. No specific products of PET or PVC cracking were identified in Samples 8–10. The bromine numbers were higher compared to the respective products of polyolefin cracking due to the presence of styrene and other unsaturated components from PS cracking. It can be concluded that small additions of PS and other plastics, like PET and PVC, promote the creation of linear components that facilitate the conversion of polyethylene. On the other hand, an addition of about 10% polystyrene to the mixture of polyethylene and polypropylene raised the PP conversion.

Hydrocarbons obtained by pyrolysis of plastics have different applications, depending on the composition. The composition of pyrolysis oil depends on both the feedstock composition and process parameters. In this study, in a continuously operated pilot tank reactor with the temperature determined by the condensable product yield, aromatic hydrocarbons were not identified for polyolefin cracking products. This means that, with the chosen conditions, feedstocks for potential use in steam cracking units can be produced. Linear hydrocarbons are the best feedstocks for ethylene production, while branched paraffins mainly yield isobutylene. Aromatics do not convert into olefins during steam cracking. It can be concluded that the best feedstock for steam cracking processes would be pyrolysis oil based on 100% PE. This product is rich in alkenes (almost 30%) which can increase coking in steam crackers; this is why it should be hydrogenated before being used in such processes. Furthermore, over 30% of the product has a boiling temperature above 350 °C, and these parts of the product should be separated. An increase of PP or PS content in the feedstock would reduce the value of the product for steam cracking processes. On the other hand, the addition of PP and PS would not affect the value of the product for other applications. After hydrogenation and proper separation, aliphatic solvents and oils with good cold temperature properties can be obtained from PP-based pyrolysis oil thanks to the high iso-paraffin content. After stabilization, the product obtained from polystyrene cracking can be a source of ethylbenzene or naphthenic components for solvents and oils. Only 3% toluene and no benzene or xylene were identified in this study, so separation of this fraction would not be feasible. These applications are feasible only if the polymer streams are pure, which means that they must come from very detailed separation processes or from post-industrial waste. In these cases, mechanical recycling would be the preferable solution. MSW consists of a mixture of these plastics, which is why chemical recycling should process streams that are not precisely separated, meaning that the mixture of PE with PP and the addition of up to 10% PS should be expected. The addition of 1% PVC or PET as contamination would not cause an issue. PET should not exceed 1% to avoid clogging of the system. This could limit the use of multilayer packaging, which can consist of a 15 micron layer of PET. Reconstruction of the cooling unit would be required to enable higher concentrations. The specified feedstock would result in a product rich in olefins, with an aromatics content proportional to the PS concentration. Such a mixture would be an excellent feedstock for the production of lubricants, solvents, oils and waxes.

## 4. Conclusions

The influence of feedstock on process parameters, yields and product composition in a continuous, pilot-scale pyrolysis process with a continuous product rate was investigated. The major conclusions are as follows:

- An increase of PP share caused decreases in the process temperature and losses yield, thus increasing product yield, and a shift of the boiling range towards lighter products;
- The addition of 10% PS to PE/PP resulted in a higher product yield without any significant changes to the boiling temperatures distribution;
- The addition of PET resulted in the creation of insoluble solids that could clog the cooling system, but mixtures with 1% PET were able to be processed;
- The addition of 5% in total of PS, PET and PVC reduced the overall product yield and caused the creation of a product with a lower boiling temperature;
- The PE cracking product consisted of 99% linear hydrocarbons, the PP cracking product consists only of branched and cyclic compounds and PS cracking product consisted of almost 98% aromatic hydrocarbons. Aromatics were not identified in either the PE or PP cracking products;
- The method used for the post-cracking analysis of the PE/PP ratio in the raw material, based on linear/branched hydrocarbons ratio, can be used for the general estimation of the feedstock composition;
- Small additions of PS and other plastics, like PET and PVC, promoted the creation of linear components, increasing the conversion of polyethylene. The addition of about 10% polystyrene to the mixture of polyethylene and polypropylene increased the PP conversion;
- The precise identification of some of the components was challenging due to the numerous types and isomers of iso-olefins obtained during polypropylene cracking and similar fragmentation of iso-olefins and cyclo-paraffins in MS analysis.

The results of this research may be helpful in the evaluation of the composition and acceptability of waste streams used for pyrolysis processes, in terms of polymer composition and optimization and the scale-up of continuous plastic cracking processes, and also for decisions regarding the further treatment or application of the obtained product.

## Figures and Tables

**Table 1:** Ratio between polymers in samples.

Sample No.	Mass Fraction, % wt.				
	PE	PP	PS	PET	PVC
1	100	0	0	0	0
2	0	100	0	0	0
3	75	25	0	0	0
4	50	50	0	0	0
5	25	75	0	0	0
6	0	0	100	0	0
7	45	45	10	0	0
8	95	0	2	2	1
9	0	95	3	1	1
10	47.5	47.5	3	1	1

**Table 2:** Cracking process temperatures and yields of products and losses.

Sample	1	2	3	4	5	6	7	8	9	10
Process temperature, °C	403	379	397	387	384	330	387	400	384	386
Product yield, % wt.	78.1	90.6	78.8	82.5	91.3	73.8	83.8	48.1	85.0	79.4
Loss (residue + gas), % wt.	21.9	9.4	21.2	17.5	8.7	26.2	16.2	51.9	15.0	20.6

**Table 3:** Boiling range distribution of condensed products. IBP - initial boiling point; FBP - final boiling point.

Fraction, % wt.	Temperature, °C									
	1	2	3	4	5	6	7	8	9	10
IBP (0.5)	80.3	91.8	77.9	78.3	82.2	71.8	79.7	79.8	75.5	81.7
10	140.0	117.8	106.1	106.1	107.1	120.0	131.9	121.4	107.1	120.8
20	193.5	172.3	159.5	157.7	166.0	130.3	162.4	171.9	166.7	177.7
30	251.1	217.0	207.9	203.9	208.9	151.4	208.0	214.8	215.8	230.6
40	287.6	243.0	264.8	250.2	232.9	221.0	244.8	270.7	271.7	285.6
50	325.1	288.9	301.2	293.1	284.7	275.1	285.6	305.9	309.9	321.5
60	356.0	316.5	336.8	328.9	316.2	284.4	322.7	341.8	344.7	356.6
70	387.7	360.3	369.8	366.6	359.3	287.7	362.4	369.1	381.9	390.1
80	419.5	395.3	407.7	402.5	396.5	344.6	399.6	402.8	417.9	424.2
90	457.3	443.5	449.1	449.0	446.2	390.6	446.6	445.4	467.2	467.7
95	485.9	479.2	480.2	481.1	480.1	421.4	479.3	474.1	500.0	498.7
FBP (99.5)	541.1	544.2	537.5	539.2	539.7	460.0	537.7	534.2	573.2	559.5

**Table 4:** Bromine numbers and group compositions of cracking products.

Sample:	1	2	3	4	5	6	7	8	9	10
Bromine numer, gBr_2_/100 g	34.20	72.80	53.27	58.22	65.82	4.25	57.22	41.62	73.0	60.18
N-paraffins	70.39	0.00	52.19	28.95	9.72	1.83	9.06	71.15	4.59	40.76
N-olefins	28.81	0.00	23.73	14.27	7.21	0.00	7.26	26.41	0.17	20.73
Iso-paraffins	0.00	6.83	5.96	7.91	7.29	0.00	3.27	0.00	3.77	2.77
Iso-olefins/cyclo-paraffins	0.80	93.17	18.12	48.87	75.27	0.42	68.08	0.00	87.06	39.28
Cyclo-olefins	0.00	0.00	0.00	0.00	0.51	0.00	0.13	0.00	0.17	0.00
Aromatics	0.00	0.00	0.00	0.00	0.00	97.75	12.20	2.44	4.24	6.46

## Data Availability

Data sharing is not applicable to this article.

## Supplementary Materials

The following are available online at https://www.mdpi.com/article/10.3390/ma14113094/s1, Table S1: PE cracking product detailed composition (Sample 1), Table S2: PP cracking product detailed composition (Sample 2), Table S3: PE/PP 75/25 cracking product detailed composition (Sample 3), Table S4: PE/PP 50/50 cracking product detailed composition (Sample 4), Table S5: PE/PP 25/75 cracking product detailed composition (Sample 5), Table S6: PS cracking product detailed composition (Sample 6), Table S7: PE/PP/PS 45/45/10 cracking product detailed composition (Sample 7), Table S8: PE/PS/PET/PVC 95/2/2/1 cracking product detailed composition (Sample 8), Table S9: PP/PS/PET/PVC 95/3/1/1 cracking product detailed composition (Sample 9), Table S10: PE/PP/PS/PET/PVC 47.5/47.5/3/1/1 cracking product detailed composition (Sample 10).
