# Supplementary material for: Influence of the Feedstock on the Process Parameters, Product Composition and Pilot-Scale Cracking of Plastics
Source: Materials (Basel). 2021 Jun 4;14(11):3094. doi: 10.3390/ma14113094 (PMC8201153; doi:10.3390/ma14113094)
Supplement: Supplementary file 1 [file materials-14-03094-s001.zip › materials-1225352-supplementary.pdf]

materials

Supplementary materials

Influence of the Feedstock on the Process Parameters, Product Composition and Pilot-Scale Cracking of Plastics

Daria Frączak [1,*], Grażyna Fabiś [1] and Beata Orlińska [2]

[1] Clariter Poland Sp. z o.o., 59A Żelazna Str., 00-848 Warszawa, Poland; info@clariter.com
[2] Department of Organic Chemical Technology and Petrochemistry, Silesian University of Technology, 4B Krzywoustego Str., 44-100 Gliwice, Poland; beata.orlinska@polsl.pl
* Correspondence: daria.fraczak@clariter.com; Tel.: +48-(512)-604-600

Table S1. PE cracking product detailed composition (Sample 1).

RT, min	%wt.	Component name	MW, Da	Structure
3.204	1.48	1-HEPTENE	98	C7H14
3.324	2.43	HEPTANE	100	C7H16
5.655	1.32	1-OCTENE	112	C8H16
5.98	2.12	OCTANE	114	C8H18
8.121	0.80	2,4-DIMETHYLHEPT-1-ENE	126	C9H18
11.527	1.16	1-NONENE	126	C9H18
12.007	1.94	NONANE	128	C9H20
16.314	2.43	1-DECENE	140	C10H20
16.634	3.01	DECANE	142	C10H22
19.73	2.54	1-UNDECENE	154	C11H22
19.975	3.72	UNDECANE	156	C11H24
22.521	2.44	1-DODECENE	168	C12H24
22.731	3.84	DODECANE	170	C12H26
24.987	2.21	1-TRIDECENE	182	C13H26
25.167	3.63	TRIDECANE	184	C13H28
27.238	2.36	1-TETRADECENE	196	C14H28
27.398	3.83	TETRADECANE	198	C14H30
29.334	1.96	1-PENTADECENE	210	C15H30
29.479	3.90	PENTADECANE	212	C15H32
31.305	1.83	CETENE	224	C16H32
31.44	4.17	HEXADECANE	226	C16H34
33.171	1.73	1-HEPTADECENE	238	C17H34
33.291	4.12	HEPTADECANE	240	C17H36
34.941	1.55	3-OCTADECENE, (E)-	252	C18H36
35.051	4.03	OCTADECANE	254	C18H38
35.126	0.36	5-OCTADECENE, (E)-	252	C18H36
36.622	1.33	1-NONADECENE	266	C19H38
36.722	3.95	NONADECANE	268	C19H40
38.228	1.10	3-EICOSENE, (E)-	280	C20H40
38.323	3.54	EICOSANE	282	C20H42
39.763	0.97	3-HENEICOSENE, (E)-	294	C21H42
39.848	3.34	HENEICOSANE	296	C21H44
41.234	0.83	9-DOCOSENE	308	C22H44
41.309	3.14	DOCOSANE	310	C22H46
42.64	0.60	1-TRICOSENE	322	C23H46
42.71	2.77	TRICOSANE	324	C23H48
43.995	0.62	9-TRICOSENE, (Z)-	322	C23H46
44.055	2.67	TETRACOSANE	338	C24H50

45.351	2.44	PENTACOSANE	352	C25H52
46.601	2.10	HEXACOSANE	366	C26H54
47.807	1.76	HEPTACOSANE	380	C27H56
48.977	1.46	OCTACOSANE	394	C28H58
50.112	1.12	NONACOSANE	408	C29H60
51.218	0.80	TRIACONTANE	422	C30H62
52.308	0.55	HENTRIACONTANE	436	C31H64
	70.39	N-PARAFFINS		
	28.81	N-OLEFINS		
	0.00	ISO-PARAFFINS		
	0.80	ISO-OLEFINS		
		CYCLO-PARAFFINS		
	0.00	CYCLO-OLEFINS		
	0.00	AROMATICS		

Table S2. PP cracking product detailed composition (Sample 2).

RT, min	%wt.	Component name	MW, Da	Structure
2.709	0.27	1-PENTENE, 2,4-DIMETHYL-	98	C7H14
2.803	0.39	2,4-DIMETHYL 1,4-PENTADIENE	96	C7H12
4.897	0.23	TRANS-1-BUTYL-2-METHYLCYCLOPROPANE	112	C8H16
5.21	1.74	HEPTANE, 4-METHYL-	114	C8H18
7.077	0.50	2,2-DIMETHYL-3-HEPTENE TRANS	126	C9H18
7.704	0.23	HEPTANE, 2,4-DIMETHYL-	128	C9H20
8.311	0.83	CYCLOHEXANE, 1,3,5-TRIMETHYL-	126	C9H18
8.365	0.40	CYCLOHEXANE, 1,2,5-TRIMETHYL-	126	C9H18
8.951	5.88	2,2-DIMETHYL-2-HEPTENE-	126	C9H18
8.985	7.76	2,4-DIMETHYL-1-HEPTENE	126	C9H18
9.992	1.38	CYCLOHEXANE, 1,4,5-TRIMETHYL-	126	C9H18
12.366	0.58	ISOPARAFFIN		
16.901	0.31	ISOOLEFIN/CYCLOPARAFFIN		
17.468	1.33	HEPTANE, 3,3,5-TRIMETHYL-	142	C10H22
17.614	1.35	HEPTANE, 3,2,5-TRIMETHYL-	142	C10H22
19.115	0.30	ISOOLEFIN/CYCLOPARAFFIN		
19.221	0.33	ISOOLEFIN/CYCLOPARAFFIN		
19.722	4.57	ISOOLEFIN/CYCLOPARAFFIN		
19.848	4.29	ISOOLEFIN/CYCLOPARAFFIN		
21.729	0.82	ISOOLEFIN/CYCLOPARAFFIN		
22.076	0.59	ISOOLEFIN/CYCLOPARAFFIN		
22.496	0.42	ISOOLEFIN/CYCLOPARAFFIN		
23.189	0.44	ISOOLEFIN/CYCLOPARAFFIN		
23.923	0.43	ISOPARAFFIN		
24.136	0.44	ISOPARAFFIN		
24.843	0.37	ISOOLEFIN/CYCLOPARAFFIN		
25.41	0.36	ISOPARAFFIN		
25.577	7.56	ISOOLEFIN/CYCLOPARAFFIN		
25.777	5.37	ISOOLEFIN/CYCLOPARAFFIN		
25.964	7.07	ISOOLEFIN/CYCLOPARAFFIN		
26.557	0.33	ISOOLEFIN/CYCLOPARAFFIN		
27.131	0.99	ISOOLEFIN/CYCLOPARAFFIN		
27.297	0.21	ISOOLEFIN/CYCLOPARAFFIN		
27.504	0.32	ISOOLEFIN/CYCLOPARAFFIN		
28.951	0.36	ISOPARAFFIN		
29.325	0.34	ISOPARAFFIN		

29.498	0.43	ISOOLEFIN/CYCLOPARAFFIN
30.172	2.23	ISOOLEFIN/CYCLOPARAFFIN
30.365	1.68	ISOOLEFIN/CYCLOPARAFFIN
30.505	0.72	ISOOLEFIN/CYCLOPARAFFIN
30.525	0.76	ISOOLEFIN/CYCLOPARAFFIN
30.719	2.04	ISOOLEFIN/CYCLOPARAFFIN
30.912	1.40	ISOOLEFIN/CYCLOPARAFFIN
31.005	0.17	ISOOLEFIN/CYCLOPARAFFIN
31.552	1.24	ISOOLEFIN/CYCLOPARAFFIN
31.726	0.24	ISOOLEFIN/CYCLOPARAFFIN
32.059	0.46	ISOOLEFIN/CYCLOPARAFFIN
34.166	2.97	ISOOLEFIN/CYCLOPARAFFIN
34.333	1.05	ISOOLEFIN/CYCLOPARAFFIN
34.487	0.86	ISOOLEFIN/CYCLOPARAFFIN
34.68	2.89	ISOOLEFIN/CYCLOPARAFFIN
34.807	0.80	ISOOLEFIN/CYCLOPARAFFIN
34.82	0.79	ISOOLEFIN/CYCLOPARAFFIN
35.047	0.76	ISOOLEFIN/CYCLOPARAFFIN
35.234	1.93	ISOOLEFIN/CYCLOPARAFFIN
35.42	1.27	ISOOLEFIN/CYCLOPARAFFIN
35.9	0.41	ISOOLEFIN/CYCLOPARAFFIN
37.728	1.35	ISOOLEFIN/CYCLOPARAFFIN
37.854	0.52	ISOOLEFIN/CYCLOPARAFFIN
37.988	0.36	ISOOLEFIN/CYCLOPARAFFIN
38.161	0.88	ISOOLEFIN/CYCLOPARAFFIN
38.301	0.47	ISOOLEFIN/CYCLOPARAFFIN
38.528	0.32	ISOOLEFIN/CYCLOPARAFFIN
38.881	1.53	ISOOLEFIN/CYCLOPARAFFIN
39.068	0.27	ISOOLEFIN/CYCLOPARAFFIN
39.288	0.37	ISOOLEFIN/CYCLOPARAFFIN
40.955	1.13	ISOOLEFIN/CYCLOPARAFFIN
41.316	1.18	ISOOLEFIN/CYCLOPARAFFIN
41.496	0.63	ISOOLEFIN/CYCLOPARAFFIN
42.016	1.88	ISOOLEFIN/CYCLOPARAFFIN
42.363	0.46	ISOOLEFIN/CYCLOPARAFFIN
42.529	0.40	ISOOLEFIN/CYCLOPARAFFIN
43.91	0.58	ISOOLEFIN/CYCLOPARAFFIN
44.223	0.39	ISOOLEFIN/CYCLOPARAFFIN
44.903	0.96	ISOOLEFIN/CYCLOPARAFFIN
46.631	0.44	ISOOLEFIN/CYCLOPARAFFIN
46.917	0.33	ISOOLEFIN/CYCLOPARAFFIN
47.564	0.77	ISOOLEFIN/CYCLOPARAFFIN
50.065	0.56	ISOOLEFIN/CYCLOPARAFFIN
	0.00	N-PARAFFINS
	0.00	N-OLEFINS
	6.83	ISO-PARAFFINS
	93.17	ISO-OLEFINS
		CYCLO-PARAFFINS
	0.00	CYCLO-OLEFINS
	0.00	AROMATICS

Table S3. PE/PP 75/25 cracking product detailed composition (Sample 3).

RT, min	%wt.	Component name	MW, Da	Structure
3.204	1.53	1-HEPTENE	98	C7H14
3.329	2.49	HEPTANE	100	C7H16
3.399	0.30	2,3-DIMETHYL-1-HEXENE	112	C8H16
4.839	2.35	HEPTANE, 4-METHYL-	114	C8H18
5.67	1.22	1-OCTENE	112	C8H16
5.99	1.83	OCTANE	114	C8H18
7.57	0.74	CYCLOHEXANE, 1,3,5-TRIMETHYL-	126	C9H18
8.026	6.04	2,4-DIMETHYL-1-HEPTENE	126	C9H18
8.996	0.84	CYCLOHEXANE, 1,2,4-TRIMETHYL-, (1.ALPHA,2.BETA,4.BETA)-	126	C9H18
11.537	1.04	1-NONENE	126	C9H18
12.017	1.61	NONANE	128	C9H20
16.314	2.07	1-DECENE	140	C10H20
16.639	2.39	DECANE	142	C10H22
16.939	0.84	OCTANE, 3,3-DIMETHYL-	142	C10H22
17.094	0.99	NONANE, 2,6-DIMETHYL-	156	C11H24
19.285	1.78	OCTANE, 2,3,6-TRIMETHYL-	156	C11H24
19.42	1.51	5-ETHYL-1-NONENE	154	C11H22
19.73	2.17	1-UNDECENE	154	C11H22
19.975	2.90	UNDECANE	156	C11H24
22.521	2.10	1-DODECENE	168	C12H24
22.731	3.04	DODECANE	170	C12H26
24.982	1.86	1-TRIDECENE	182	C13H26
25.167	2.94	TRIDECANE	184	C13H28
25.227	2.67	1-UNDECENE, 7-METHYL-	168	C12H24
25.422	1.09	1-UNDECENE, 4-METHYL-	168	C12H24
25.612	2.31	1-UNDECENE, 4-METHYL-	168	C12H24
27.233	2.00	1-TETRADECENE	196	C14H28
27.393	2.97	TETRADECANE	198	C14H30
29.329	1.63	1-PENTADECENE	210	C15H30
29.474	3.00	PENTADECANE	212	C15H32
29.849	0.59	CYCLOHEXANE, 2,4-DIISOPROPYL-1,1-DIMETHY	196	C14H28
30.389	0.35	CYCLOHEXANE, 1,5-DIISOPROPYL-2,3-DIMETHY	196	C14H28
31.3	1.52	CETENE	224	C16H32
31.435	3.09	HEXADECANE	226	C16H34
33.166	1.43	1-HEPTADECENE	238	C17H34
33.286	2.84	HEPTADECANE	240	C17H36
34.351	0.49	ISOOLEFIN/CYCLOPARAFFIN		
34.936	1.39	3-OCTADECENE, (E)-	252	C18H36
35.046	2.74	OCTADECANE	254	C18H38
35.116	0.44	ISOOLEFIN/CYCLOPARAFFIN		
36.617	1.08	1-NONADECENE	266	C19H38
36.722	2.67	NONADECANE	268	C19H40
38.223	0.95	3-EICOSENE, (E)-	280	C20H40
38.318	2.49	EICOSANE	282	C20H42
39.758	0.76	3-HENEICOSENE, E)-	294	C21H42
39.843	2.34	HENEICOSANE	296	C21H44
41.229	0.72	9-DOCOSENE	308	C22H44
41.304	2.09	DOCOSANE	310	C22H46
42.639	0.45	1-TRICOSENE	322	C23H46
42.704	1.86	TRICOSANE	324	C23H48
43.99	0.57	1-TETRACOSENE	336	C24H48

44.055	1.88	TETRACOSANE	338	C24H50
45.35	1.67	PENTACOSANE	352	C25H52
46.601	1.48	HEXACOSANE	366	C26H54
47.806	1.22	HEPTACOSANE	380	C27H56
48.977	0.99	OCTACOSANE	394	C28H58
50.117	0.75	NONACOSANE	408	C29H60
51.223	0.53	TRIACONTANE	422	C30H62
52.313	0.37	HENTRIACONTANE	436	C31H64
	52.18	N-PARAFFINS		
	23.73	N-OLEFINS		
	5.96	ISO-PARAFFINS		
	18.12	ISO-OLEFINS		
		CYCLO-PARAFFINS		
	0.00	CYCLO-OLEFINS		
	0.00	AROMATICS		

Table S4. PE/PP 50/50 cracking product detailed composition (Sample 4).

RT, min	%wt.	Component name	MW, Da	Structure
3.204	0.91	1-HEPTENE	98	C7H14
3.324	1.26	HEPTANE	100	C7H16
3.394	0.33	2,3-DIMETHYL-1-HEXENE	112	C8H16
4.589	0.36	2-HEXENE, 2,5-DIMETHYL-	112	C8H16
4.819	2.91	HEPTANE, 4-METHYL-	114	C8H18
5.66	0.69	1-OCTENE	112	C8H16
5.985	0.98	OCTANE	114	C8H18
6.49	0.33	CYCLOHEXANE, 1,1,2-TRIMETHYL-	126	C9H18
7.09	0.25	1-HEXENE, 3, 5, 5-TRIMETHYL-	126	C9H18
7.53	0.73	CYCLOHEXANE, 1,3,5-TRIMETHYL-, (1.ALPHA, 3.ALPHA,5.BETA)-	126	C9H18
7.955	8.57	2,4-DIMETHYL-1-HEPTENE	126	C9H18
8.946	1.17	CYCLOHEXANE, 1,3,5-TRIMETHYL-	126	C9H18
11.537	0.54	1-NONENE	126	C9H18
11.662	0.36	1-UNDECENE, 8-METHYL-	168	C12H24
12.022	0.83	NONANE	128	C9H20
16.319	0.98	1-DECENE	140	C10H20
16.644	1.09	DECANE	142	C10H22
16.939	1.52	NONANE, 2,6-DIMETHYL-	156	C11H24
17.094	1.68	OCTANE, 2,3,7-TRIMETHYL-	156	C11H24
19.29	3.36	2-DECENE, 2,4-DIMETHYL-	168	C12H24
19.425	2.91	1-DECENE, 2,4-DIMEYTHYL-	168	C12H24
19.74	1.09	1-UNDECENE	154	C11H22
19.985	1.39	UNDECANE	156	C11H24
20.726	0.23	1-UNDECENE, 9-METHYL-	168	C12H24
21.381	0.57	BICYCLO[3.1.1]HEPTANE,2,6,6-TRIMETHYL-,(1.ALPHA,2.BETA,5.ALPHA)-	138	C10H18
21.456	0.29	(-)-TRANS-PINANE	138	C10H18
21.706	0.23	1,3-DIISOPROPYL CYCLOHEXANE	168	C12H24
22.531	1.10	1-DODECENE	168	C12H24
22.741	1.49	DODECANE	170	C12H26
23.577	0.40	DECANE, 2,3,5,8-TETRAMETHYL-	198	C14H30
23.782	0.41	DODCECANE, 4,6-DIMETHYL-	198	C14H30
23.952	0.32	ISOOLEFIN/CYCLOPARAFFIN		
24.997	1.06	1-TRIDECENE	182	C13H26
25.182	1.51	TRIDECANE	184	C13H28
25.237	5.31	1-UNDECENE, 7-METHYL-	168	C12H24

25.437	2.75	ISOOLEFIN/CYCLOPARAFFIN		
25.628	4.63	ISOOLEFIN/CYCLOPARAFFIN		
26.238	0.28	ISOOLEFIN/CYCLOPARAFFIN		
26.828	0.59	ISOOLEFIN/CYCLOPARAFFIN		
27.243	1.15	1-TETRADECENE	196	C14H28
27.408	1.51	TETRADECANE	198	C14H30
28.619	0.38	DODECANE, 2,6,11-TRIMETHYL-	212	C15H32
28.994	0.28	DODECANE, 4,6-DIMETHYL-	198	C14H30
29.184	0.33	DECANE, 3,3,5-TRIMETHYL-	184	C13H28
29.339	0.97	1-PENTADECENE	210	C15H30
29.484	1.65	PENTADECANE	212	C15H32
29.859	1.40	ISOOLEFIN/CYCLOPARAFFIN		
30.054	0.82	ISOOLEFIN/CYCLOPARAFFIN		
30.199	0.31	ISOOLEFIN/CYCLOPARAFFIN		
30.399	1.04	ISOOLEFIN/CYCLOPARAFFIN		
30.599	0.58	ISOOLEFIN/CYCLOPARAFFIN		
31.255	0.59	ISOOLEFIN/CYCLOPARAFFIN		
31.31	0.88	CETENE	224	C16H32
31.445	1.69	HEXADECANE	226	C16H34
31.76	0.26	ISOOLEFIN/CYCLOPARAFFIN		
33.176	0.82	1-HEPTADECENE	238	C17H34
33.301	1.59	HEPTADECANE	240	C17H36
33.851	1.83	ISOOLEFIN/CYCLOPARAFFIN		
34.016	0.39	ISOOLEFIN/CYCLOPARAFFIN		
34.166	0.30	ISOOLEFIN/CYCLOPARAFFIN		
34.361	1.58	ISOOLEFIN/CYCLOPARAFFIN		
34.491	0.23	ISOOLEFIN/CYCLOPARAFFIN		
34.726	0.25	ISOOLEFIN/CYCLOPARAFFIN		
34.931	1.20	3-OCTADECENE, (E)-	252	C18H36
35.056	1.58	OCTADECANE	254	C18H38
35.121	0.68	ISOOLEFIN/CYCLOPARAFFIN		
36.632	0.69	1-NONADECENE	266	C19H38
36.732	1.47	NONADECANE	268	C19H40
37.412	0.81	ISOOLEFIN/CYCLOPARAFFIN		
37.542	0.22	ISOOLEFIN/CYCLOPARAFFIN		
37.837	0.41	ISOOLEFIN/CYCLOPARAFFIN		
38.233	0.60	3-EICOSENE, (E)-	280	C20H40
38.323	1.41	EICOSANE	282	C20H42
38.573	0.70	ISOOLEFIN/CYCLOPARAFFIN		
39.768	0.46	3-HENEICOSENE, (E)-	294	C21H42
39.853	1.26	HENEICOSANE	296	C21H44
40.633	0.62	ISOOLEFIN/CYCLOPARAFFIN		
40.984	0.46	ISOOLEFIN/CYCLOPARAFFIN		
41.239	0.38	9-EICOSENE, (E)-	280	C20H40
41.314	1.21	DOCOSANE	310	C22H46
41.699	0.63	ISOOLEFIN/CYCLOPARAFFIN		
42.649	0.37	1-DOCOSENE	308	C22H44
42.714	1.22	TRICOSANE	324	C23H48
43.565	0.35	ISOOLEFIN/CYCLOPARAFFIN		
44.005	0.38	1-TRICOSENE	322	C23H46
44.06	1.10	TETRACOSANE	338	C24H50
44.565	0.34	ISOOLEFIN/CYCLOPARAFFIN		
45.355	1.05	PENTACOSANE	352	C25H52
46.261	0.25	ISOOLEFIN/CYCLOPARAFFIN		

46.611	0.92	HEXACOSANE	366	C26H54
47.196	0.27	ISOOLEFIN/CYCLOPARAFFIN		
47.816	0.80	HEPTACOSANE	380	C27H56
48.987	0.70	OCTACOSANE	394	C28H58
50.117	0.55	NONACOSANE	408	C29H60
51.223	0.41	TRIACONTANE	422	C30H62
52.308	0.28	HENTRIACONTANE	436	C31H64
	28.95	N-PARAFFINS		
	14.27	N-OLEFINS		
	7.91	ISO-PARAFFINS		
	48.87	ISO-OLEFINS		
		CYCLO-PARAFFINS		
	0.00	CYCLO-OLEFINS		
	0.00	AROMATICS		

Table S5. PE/PP 25/75 cracking product detailed composition (Sample 5).

RT, min	%wt.	Component name	MW, Da	Structure
3.189	0.39	1-HEPTENE	98	C7H14
4.564	0.44	CYCLOPENTANE, 1,2,4-TRIMETHYL-	112	C8H16
4.784	2.74	HEPTANE, 4-METHYL-	114	C8H18
5.965	0.20	OCTANE	114	C8H18
6.44	0.28	CYCLOHEXANE, 1,1,2-TRIMETHYL-	126	C9H18
7.05	0.16	HEPTANE, 2,4-DIMETHYL-	128	C9H20
7.46	0.78	CYCLOHEXANE, 1,3,5-TRIMETHYL-	126	C9H18
7.895	11.20	2,4-DIMETHYL-1-HEPTENE	126	C9H18
8.861	1.30	CYCLOHEXANE, 1,2,4-TRIMETHYL-, (1.ALPHA,2.BETA,4.BETA)-	126	C9H18
10.051	0.12	CYCLOHEXENE, 3,3,5-TRIMETHYL	124	C9H16
11.567	0.38	1-NONENE	126	C9H18
11.987	0.15	PENTANE, 2,2,3,4-TETRAMETHYL-	128	C9H20
16.299	0.34	1-DECENE	140	C10H20
16.359	0.19	CYCLOPROPANE, 1,2-DIMETHYL-1-PENTYL-	140	C10H20
16.624	0.29	DECANE	142	C10H22
16.914	1.71	NONANE, 2,6-DIMETHYL-	156	C11H24
17.069	1.85	OCTANE, 2,3,6-TRIMETHYL-	156	C11H24
18.68	0.22	ISOOLEFIN/CYCLOPARAFFIN		
18.775	0.22	1-NONENE, 4,6,8-TRIMETHYL-	168	C12H24
19.275	4.53	1-UNDECENE, 5-METHYL-	168	C12H24
19.41	4.34	1-DECENE, 2,4-DIMETHYL-	168	C12H24
19.72	0.35	1-UNDECENE	154	C11H22
19.97	0.41	UNDECANE	156	C11H24
21.361	0.72	BICYCLO[3.1.1]HEPTANE,2,6,6-TRIMETHYL-,(1.ALPHA,2.BETA,5.ALPHA)-	138	C10H18
21.436	0.43	(-)-TRANS-PINANE	138	C10H18
22.516	0.36	1,3-DIISOPROPYL CYCLOHEXANE	168	C12H24
22.726	0.43	DODECANE	170	C12H26
23.562	0.34	DECANE, 2,3,5,8-TETRAMETHYL-	198	C14H30
23.767	0.34	DODCECANE, 4,6-DIMETHYL-	198	C14H30
24.982	0.33	ISOOLEFIN/CYCLOPARAFFIN		
25.237	7.99	ISOOLEFIN/CYCLOPARAFFIN		
25.432	4.97	ISOOLEFIN/CYCLOPARAFFIN		
25.628	7.45	ISOOLEFIN/CYCLOPARAFFIN		
26.818	0.87	ISOOLEFIN/CYCLOPARAFFIN		
27.233	0.45	1-TETRADECENE	196	C14H28
27.398	0.50	TETRADECANE	198	C14H30

29.334	0.43	1-PENTADECENE	210	C15H30
29.479	0.64	PENTADECANE	210	C15H32
29.854	2.30	ISOOLEFIN/CYCLOPARAFFIN		
30.049	1.48	ISOOLEFIN/CYCLOPARAFFIN		
30.189	0.56	ISOOLEFIN/CYCLOPARAFFIN		
30.394	1.88	ISOOLEFIN/CYCLOPARAFFIN		
30.594	0.94	ISOOLEFIN/CYCLOPARAFFIN		
31.25	1.13	ISOOLEFIN/CYCLOPARAFFIN		
31.31	0.45	CETENE	224	C16H32
31.44	0.74	HEXADECANE	226	C16H34
32.54	0.13	ISOOLEFIN/CYCLOPARAFFIN		
33.175	0.44	1-HEPTADECENE	238	C17H34
33.296	0.60	HEPTADECANE	240	C17H36
33.851	3.06	ISOOLEFIN/CYCLOPARAFFIN		
34.016	0.84	ISOOLEFIN/CYCLOPARAFFIN		
34.166	0.50	ISOOLEFIN/CYCLOPARAFFIN		
34.366	3.19	ISOOLEFIN/CYCLOPARAFFIN		
34.491	0.67	ISOOLEFIN/CYCLOPARAFFIN		
34.731	0.39	ISOOLEFIN/CYCLOPARAFFIN		
34.926	1.73	3-OCTADECENE, (E)-	252	C18H36
35.056	0.85	OCTADECANE	254	C18H38
35.121	1.19	ISOOLEFIN/CYCLOPARAFFIN		
36.632	0.44	1-NONADECENE	266	C19H38
36.737	0.60	NONADECANE	268	C19H40
37.417	1.37	ISOOLEFIN/CYCLOPARAFFIN		
37.542	0.43	ISOOLEFIN/CYCLOPARAFFIN		
37.842	0.66	ISOOLEFIN/CYCLOPARAFFIN		
38.243	0.42	3-EICOSENE, (E)-	280	C20H40
38.338	0.59	EICOSANE	282	C20H42
38.578	1.32	ISOOLEFIN/CYCLOPARAFFIN		
39.778	0.35	HENEICOS-1-ENE	294	C21H42
39.863	0.55	HENEICOSANE	296	C21H44
40.644	1.02	ISOOLEFIN/CYCLOPARAFFIN		
40.749	0.29	ISOOLEFIN/CYCLOPARAFFIN		
40.999	0.88	ISOOLEFIN/CYCLOPARAFFIN		
41.169	0.38	ISOOLEFIN/CYCLOPARAFFIN		
41.259	0.42	9-EICOSENE, (E)-	280	C20H40
41.334	0.63	DOCOSANE	310	C22H46
41.714	1.42	ISOOLEFIN/CYCLOPARAFFIN		
41.824	0.26	ISOOLEFIN/CYCLOPARAFFIN		
42.674	0.30	1-DOCOSENE	308	C22H44
42.744	0.53	TRICOSANE	324	C23H48
43.59	0.50	ISOOLEFIN/CYCLOPARAFFIN		
43.91	0.31	1-TRICOSENE	322	C23H46
44.105	0.51	TETRACOSANE	338	C24H50
44.595	0.85	ISOOLEFIN/CYCLOPARAFFIN		
45.05	0.40	1,3,5-TRIS(CYCLOHEXYL)PENT-1-ENE	316	C23H40
45.41	0.42	PENTACOSANE	352	C25H52
46.681	0.51	HEXACOSANE	366	C26H54
47.236	0.48	ISOOLEFIN/CYCLOPARAFFIN		
47.906	0.32	HEPTACOSANE	380	C27H56
48.802	0.20	ISOOLEFIN/CYCLOPARAFFIN		
49.127	0.28	OCTACOSANE	394	C28H58
49.692	0.22	ISOOLEFIN/CYCLOPARAFFIN		

50.277	0.14	NONACOSANE	408	C29H60
51.998	0.16	ISOOLEFIN/CYCLOPARAFFIN		
	9.72	N-PARAFFINS		
	7.21	N-OLEFINS		
	7.29	ISO-PARAFFINS		
	75.27	ISO-OLEFINS		
		CYCLO-PARAFFINS		
	0.51	CYCLO-OLEFINS		
	0.00	AROMATICS		

Table S6. PS cracking product detailed composition (Sample 6).

RT, min	%wt.	Component name	MW, Da	Structure
4.829	3.03	TOLUENE	92	C7H8
8.126	0.42	2,4-DIMETHYL-1-HEPTENE	126	C9H18
9.146	6.30	ETHYLBENZENE	106	C8H10
11.427	33.90	STYRENE	104	C8H8
13.178	1.49	BENZENE, (1-METHYLETHYL)-	120	C9H12
15.899	7.75	.ALPHA.-METHYLSTYRENE	118	C9H10
27.403	0.36	TETRADECANE	198	C14H30
29.484	0.36	PENTADECANE	212	C15H32
31.445	0.36	HEXADECANE	226	C16H34
32.71	18.13	BENZENE, 1,1'-(1,3-PROPANEDIYL)BIS-	196	C15H16
33.16	2.15	BENZENE, 1,1'-(1-METHYL-1,3-PROPANEDIYL)	210	C16H18
33.321	0.40	HEPTADECANE	240	C17H36
33.916	12.13	BENZENE, 1,1'-(2-BUTENE-1,4-DIYL)BIS-	208	C16H16
34.011	2.78	BENZENE, 1,1'-(2-PENTENE-1,5-DIYL)BIS-	222	C17H18
34.186	1.80	1,2-DIPHENYLCYCLOPROPANE	194	C15H14
35.056	0.35	OCTADECANE	254	C18H38
35.286	0.35	BENZENE, 1,1'-(3-METHYL-1-PROPENE-1,3-DIYL)BIS-	208	C16H16
44.92	7.32	BENZENE, 1,1'-[1-(2,2-DIMETHYL-3-BUTENYL)-1,3-PROPANEDIYL]BIS-	278	C21H26
51.023	0.62	1,1':3',1''-TERPHENYL, 5'-PHENYL-	306	C24H18
	1.83	N-PARAFFINS		
	0.00	N-OLEFINS		
	0.00	ISO-PARAFFINS		
	0.42	ISO-OLEFINS		
		CYCLO-PARAFFINS		
	0.00	CYCLO-OLEFINS		
	97.75	AROMATICS		

Table S7. PE/PP/PS 45/45/10 cracking product detailed composition (Sample 7).

RT, min	%wt.	Component name	MW, Da	Structure
3.174	0.21	1-HEPTENE	98	C7H14
3.294	0.30	HEPTANE	100	C7H16
4.544	0.18	2,3-DIMETHYL-1-HEXENE	112	C8H16
4.784	4.80	TOLUENE	92	C7H8
5.62	0.23	OCTENE	112	C8H16
5.94	0.24	OCTANE	114	C8H18
6.405	0.27	1-HEXENE, 3,3,5-TRIMETHYL-	126	C9H18
7.04	0.18	CYCLOHEXANE, 1,2,4-TRIMETHYL-	126	C9H18
7.445	0.62	CYCLOHEXANE, 1,3,5-TRIMETHYL-	126	C9H18
7.87	7.93	2,4-DIMETHYL-1-HEPTENE	126	C9H18
8.836	1.13	CYCLOHEXANE, 1,3,5-TRIMETHYL-	126	C9H18

9.121	0.90	ETHYLBENZENE	106	C8H10
9.991	0.14	5-METHYLOCTENE-1	126	C9H18
10.632	0.13	CYCLOHEXENE, 3,3,5-TRIMETHYL-	124	C9H16
11.267	3.12	STYRENE	104	C8H8
11.517	0.68	1-NONENE	126	C9H18
11.967	0.27	NONANE	128	C9H20
13.153	0.14	BENZENE, (1-METHYLETHYL)-	120	C9H12
15.839	0.89	.ALPHA.-METHYLSTYRENE	118	C9H10
16.279	0.30	1-DECENE	140	C10H20
16.609	0.26	DECANE	142	C10H22
16.894	1.60	NONANE, 2,6-DIMETHYL-	156	C11H24
17.049	1.67	NONANE, 2,4-DIMETHYL-	156	C11H24
18.585	0.25	2-HEPTENE, 5-ETHYL-2,4-DIMETHYL	154	C11H22
18.655	0.40	2-DECENE, 2,4-DIMETHYL-	168	C12H24
18.75	0.41	5-ETHYL-1-NONENE	154	C11H22
19.25	3.53	2-DECENE, 8-METHYL-, (Z)-	154	C11H22
19.39	3.11	2-DECENE, 8-METHYL-, (Z)-	154	C11H22
19.705	0.31	UNDECENE	154	C11H22
19.95	0.40	UNDECANE	156	C11H24
21.346	0.55	2-DECENE, 2,4-DIMETHYL-	168	C12H24
21.416	0.29	1-ISOPROPYL-1,4,5-TRIMETHYLCYCLOHEXANE	168	C12H24
22.501	0.33	1,3-DIISOPROPYL CYCLOHEXANE	168	C12H24
22.646	0.14	1-DODECENE	168	C12H24
22.706	0.44	DODECANE	170	C12H26
23.547	0.30	ISOOLEFIN/CYCLOPARAFFIN	182	C13H26
24.967	0.33	1-TRIDECENE	182	C13H26
25.062	0.47	TRIDECANE	184	C13H28
25.217	6.97	1-UNDECENE, 7-METHYL-	168	C12H24
25.413	3.74	ISOOLEFIN/CYCLOPARAFFIN	196	C14H28
25.603	6.12	ISOOLEFIN/CYCLOPARAFFIN	196	C14H28
25.673	1.05	BENZENE, HEPTYL-	176	C13H20
26.093	0.24	ISOOLEFIN/CYCLOPARAFFIN	196	C14H28
26.213	0.53	ISOOLEFIN/CYCLOPARAFFIN	196	C14H28
26.708	0.49	ISOOLEFIN/CYCLOPARAFFIN	196	C14H28
26.803	1.04	ISOOLEFIN/CYCLOPARAFFIN	196	C14H28
26.898	0.20	1-CYCLOPENTYL-4-(1-METHYLETHYL)CYCLOHEXANE	194	C14H26
27.223	0.59	1-TETRADECENE	196	C14H28
27.378	0.40	TETRADECANE	198	C14H30
29.839	2.03	ISOOLEFIN/CYCLOPARAFFIN	226	C15H30
30.029	1.31	ISOOLEFIN/CYCLOPARAFFIN	226	C15H30
30.379	1.49	ISOOLEFIN/CYCLOPARAFFIN	226	C15H30
30.575	0.83	ISOOLEFIN/CYCLOPARAFFIN	212	C15H32
30.67	0.22	ISOOLEFIN/CYCLOPARAFFIN	226	C16H34
31.155	0.21	ISOOLEFIN/CYCLOPARAFFIN	226	C16H34
31.235	1.02	ISOOLEFIN/CYCLOPARAFFIN	226	C16H34
31.29	0.64	CETENE	224	C16H32
31.42	0.75	HEXADECANE	226	C16H34
32.53	0.52	ISOOLEFIN/CYCLOPARAFFIN	238	C17H34
32.61	1.31	BENZENE, 1,1'-(1,4-BUTANEDIYL)BIS-	210	C16H18
32.705	0.25	ISOOLEFIN/CYCLOPARAFFIN	238	C17H34
33.061	0.27	ISOOLEFIN/CYCLOPARAFFIN	238	C17H34
33.161	0.51	1-HEPTADECENE	238	C17H34
33.281	0.64	HEPTADECANE	240	C17H36
33.831	4.02	ISOOLEFIN/CYCLOPARAFFIN	252	C18H36

33.996	0.51	ISOOLEFIN/CYCLOPARAFFIN	252	C18H36
34.146	0.55	ISOOLEFIN/CYCLOPARAFFIN	252	C18H36
34.346	2.81	ISOOLEFIN/CYCLOPARAFFIN	252	C18H36
34.476	0.57	ISOOLEFIN/CYCLOPARAFFIN	252	C18H36
34.906	1.50	3-OCTADECENE, (E)-	252	C18H36
35.041	0.78	OCTADECANE	252	C18H38
35.101	1.12	ISOOLEFIN/CYCLOPARAFFIN	266	C19H38
35.577	0.43	ISOOLEFIN/CYCLOPARAFFIN	266	C19H38
36.617	0.41	1-NONADECENE	266	C19H38
36.717	0.46	NONADECANE	266	C19H40
37.402	1.39	ISOOLEFIN/CYCLOPARAFFIN	280	C20H40
37.527	0.42	ISOOLEFIN/CYCLOPARAFFIN	280	C20H40
37.827	0.51	ISOOLEFIN/CYCLOPARAFFIN	280	C20H40
38.233	0.45	EICOSENE	280	C20H40
38.318	0.36	EICOSANE	282	C20H42
38.563	0.97	ISOOLEFIN/CYCLOPARAFFIN	294	C21H42
39.763	0.29	HENEICOSENE	294	C21H42
39.848	0.47	HENEICOSANE	296	C21H44
40.629	0.99	ISOOLEFIN/CYCLOPARAFFIN	308	C22H44
40.729	0.35	ISOOLEFIN/CYCLOPARAFFIN	308	C22H44
40.984	0.69	ISOOLEFIN/CYCLOPARAFFIN	308	C22H44
41.159	0.33	ISOOLEFIN/CYCLOPARAFFIN	308	C22H44
41.249	0.44	9-DOCOSENE	308	C22H44
41.314	0.51	DOCOSANE	310	C22H46
41.694	1.04	ISOOLEFIN/CYCLOPARAFFIN	308	C22H44
42.659	0.25	TRICOSENE	322	C23H46
42.729	0.40	TRICOSANE	324	C23H48
43.575	0.51	ISOOLEFIN/CYCLOPARAFFIN	336	C24H48
43.67	0.21	ISOOLEFIN/CYCLOPARAFFIN	336	C24H48
43.895	0.31	ISOOLEFIN/CYCLOPARAFFIN	336	C24H48
43.98	0.61	ISOOLEFIN/CYCLOPARAFFIN	336	C24H48
44.09	0.52	TETRACOSANE	338	C24H50
44.58	0.56	ISOOLEFIN/CYCLOPARAFFIN	350	C25H50
44.715	0.30	ISOOLEFIN/CYCLOPARAFFIN	350	C25H50
45.33	0.35	PENTACOSANE	352	C25H52
46.276	0.41	ISOOLEFIN/CYCLOPARAFFIN	364	C26H52
46.566	0.29	ISOOLEFIN/CYCLOPARAFFIN	364	C26H52
46.656	0.40	HEXACOSANE	366	C26H54
47.126	0.21	ISOOLEFIN/CYCLOPARAFFIN	378	C27H54
47.216	0.42	ISOOLEFIN/CYCLOPARAFFIN	378	C27H54
47.881	0.24	HEPTACOSANE	380	C27H56
48.787	0.23	ISOOLEFIN/CYCLOPARAFFIN		
49.112	0.26	OCTACOSANE	394	C28H58
49.667	0.21	ISOOLEFIN/CYCLOPARAFFIN		
50.262	0.15	NONACOSANE	408	C29H60
	9.06	N-PARAFFINS		
	7.27	N-OLEFINS		
	3.27	ISO-PARAFFINS		
	68.08	ISO-OLEFINS		
		CYCLO-PARAFFINS		
	0.13	CYCLO-OLEFINS		
	12.20	AROMATICS		

Table S8. PE/PS/PET/PVC 95/2/2/1 cracking product detailed composition (Sample 8).

RT, min	%wt.	Component name	MW, Da	Structure
3.204	1.20	1-HEPTENE	98	C7H14
3.324	2.10	HEPTANE	100	C7H16
4.844	1.18	TOLUENE	92	C7H8
5.66	1.29	1-OCTENE	112	C8H16
5.98	1.99	OCTANE	114	C8H18
9.251	0.39	ETHYLBENZENE	106	C8H10
11.402	0.43	STYRENE	104	C8H8
11.532	1.02	1-NONENE	126	C9H18
12.012	1.78	NONANE	128	C9H20
16.314	2.07	1-DECENE	140	C10H20
16.634	2.83	DECANE	142	C10H22
19.73	2.25	1-UNDECENE	154	C11H22
19.975	3.65	UNDECANE	156	C11H24
22.521	2.27	1-DODECENE	168	C12H24
22.732	3.95	DODECANE	170	C12H26
24.987	2.12	1-TRIDECENE	182	C13H26
25.168	4.05	TRIDECANE	184	C13H28
27.233	2.34	1-TETRADECENE	196	C14H28
27.398	4.16	TETRADECANE	198	C14H30
29.329	1.87	1-PENTADECENE	210	C15H30
29.479	4.33	PENTADECANE	212	C15H32
31.3	1.72	CETENE	224	C16H32
31.435	4.40	HEXADECANE	226	C16H34
33.166	1.59	1-HEPTADECENE	238	C17H34
33.286	4.27	HEPTADECANE	240	C17H36
33.831	0.44	BENZENE, 1,1'-(2-BUTENE-1,4-DIYL)BIS-	208	C16H16
34.936	1.41	3-OCTADECENE, (E)-	252	C18H36
35.047	4.09	OCTADECANE	254	C18H38
35.127	0.33	5-OCTADECENE, (E)-	252	C18H36
36.622	1.20	1-NONADECENE	266	C19H38
36.722	4.01	NONADECANE	268	C19H40
38.228	1.01	3-EICOSENE, (E)-	280	C20H40
38.318	3.66	EICOSANE	282	C20H42
39.763	0.91	3-HENEICOSENE, (E)-	294	C21H42
39.843	3.47	HENEICOSANE	296	C21H44
41.229	0.74	9-DOCOSENE	308	C22H44
41.304	3.18	DOCOSANE	310	C22H46
42.64	0.54	1-TRICOSENE	322	C23H46
42.705	2.91	TRICOSANE	324	C23H48
43.985	0.53	9-TRICOSENE, (Z)-	322	C23H46
44.05	2.59	TETRACOSANE	338	C24H50
45.346	2.45	PENTACOSANE	352	C25H52
46.596	2.06	HEXACOSANE	366	C26H54
47.807	1.71	HEPTACOSANE	380	C27H56
48.977	1.34	OCTACOSANE	394	C28H58
50.113	0.99	NONACOSANE	408	C29H60
51.218	0.72	TRIACONTANE	422	C30H62
52.304	0.46	HENTRIACONTANE	436	C31H64
	71.15	N-PARAFFINS		
	26.41	N-OLEFINS		
	0.00	ISO-PARAFFINS		
	0.00	ISO-OLEFINS		
	0.00	CYCLO-PARAFFINS		

	0.00	CYCLO-OLEFINS		
	2.45	AROMATICS		

Table S9: PP/PS/PET/PVC 95/3/1/1 cracking product detailed composition (Sample 9)

RT, min	%wt.	Component name	MW, Da	Structure
3.389	0.51	2,3-DIMETHYL-1-HEXENE	112	C8H16
4.554	0.59	2-HEXENE, 2,5-DIMETHYL-	112	C8H16
4.799	2.52	TOLUENE	92	C7H8
6.485	0.20	CYCLOHEXANE, 1,1,2-TRIMETHYL-	126	C9H18
7.515	2.90	2,3-DIMETHYL-2-HEPTENE	126	C9H18
7.915	9.52	2,4-DIMETHYL-1-HEPTENE	126	C9H18
8.901	0.91	CYCLOHEXANE, 1,3,5-TRIMETHYL-	126	C9H18
9.211	0.29	ETHYLBENZENE	106	C8H10
10.066	0.17	CYCLOHEXENE, 3,3,5-TRIMETHYL-	124	C9H16
11.332	1.28	STYRENE	104	C8H8
11.612	0.37	1-UNDECENE, 8-METHYL-	168	C12H24
15.894	0.15	.ALPHA.-METHYLSTYRENE	118	C9H10
16.929	1.50	NONANE, 2,6-DIMETHYL-	156	C11H24
17.084	1.54	OCTANE, 2,3,7-TRIMETHYL-	156	C11H24
18.685	1.31	2-OCTENE, 2,3,7-TRIMETHYL-	154	C11H22
18.785	1.22	2-DECENE, 2,2-DIMETHYL-	168	C12H24
19.285	3.56	2-DECENE, 2,4-DIMETHYL-	168	C12H24
19.42	3.33	1-DECENE, 2,4-DIMEYTHYL-	168	C12H24
20.571	0.26	1,6-OCTADIENE, 5,7-DIMETHYL-,(R)-	138	C10H18
21.376	0.44	ISOOLEFIN/CYCLOPARAFFIN		
21.446	0.23	ISOOLEFIN/CYCLOPARAFFIN		
21.701	0.16	ISOOLEFIN/CYCLOPARAFFIN		
23.572	0.25	DECANE, 2,3,5,8-TETRAMETHYL-	198	C14H30
23.782	0.27	DODCECANE, 4,6-DIMETHYL-	198	C14H30
24.522	3.04	ISOOLEFIN/CYCLOPARAFFIN		
24.687	0.89	ISOOLEFIN/CYCLOPARAFFIN		
24.847	0.80	ISOOLEFIN/CYCLOPARAFFIN		
25.087	2.71	ISOOLEFIN/CYCLOPARAFFIN		
25.237	6.64	1-UNDECENE, 7-METHYL-	168	C12H24
25.438	4.07	ISOOLEFIN/CYCLOPARAFFIN		
25.628	5.94	ISOOLEFIN/CYCLOPARAFFIN		
26.048	0.15	CYCLOHEXANE, 2,4-DIETHYL-1-METHYL-	154	C11H22
26.118	0.52	ISOOLEFIN/CYCLOPARAFFIN		
26.238	0.38	ISOOLEFIN/CYCLOPARAFFIN		
26.728	0.26	ISOOLEFIN/CYCLOPARAFFIN		
26.823	0.46	ISOOLEFIN/CYCLOPARAFFIN		
28.619	0.22	DODECANE, 2,6,11-TRIMETHYL-	212	C15H32
29.184	0.92	ISOOLEFIN/CYCLOPARAFFIN		
29.344	0.41	ISOOLEFIN/CYCLOPARAFFIN		
29.484	0.57	PENTADECANE	212	C15H32
29.589	0.25	ISOOLEFIN/CYCLOPARAFFIN		
29.714	0.40	ISOOLEFIN/CYCLOPARAFFIN		
29.859	1.50	ISOOLEFIN/CYCLOPARAFFIN		
30.054	1.21	ISOOLEFIN/CYCLOPARAFFIN		
30.184	0.48	ISOOLEFIN/CYCLOPARAFFIN		
30.399	1.27	ISOOLEFIN/CYCLOPARAFFIN		
30.605	1.13	ISOOLEFIN/CYCLOPARAFFIN		
31.25	0.54	ISOOLEFIN/CYCLOPARAFFIN		
31.44	0.36	HEXADECANE	226	C16H34

33.181	0.27	ISOOLEFIN/CYCLOPARAFFIN		
33.251	1.25	ISOOLEFIN/CYCLOPARAFFIN		
33.386	0.31	ISOOLEFIN/CYCLOPARAFFIN		
33.696	0.98	ISOOLEFIN/CYCLOPARAFFIN		
33.856	2.31	ISOOLEFIN/CYCLOPARAFFIN		
34.021	0.73	ISOOLEFIN/CYCLOPARAFFIN		
34.361	2.20	ISOOLEFIN/CYCLOPARAFFIN		
34.431	0.79	ISOOLEFIN/CYCLOPARAFFIN		
34.526	0.83	ISOOLEFIN/CYCLOPARAFFIN		
34.921	1.22	ISOOLEFIN/CYCLOPARAFFIN		
35.056	0.52	OCTADECANE	254	C18H38
35.116	0.51	ISOOLEFIN/CYCLOPARAFFIN		
36.732	0.36	ISOOLEFIN/CYCLOPARAFFIN		
36.857	0.52	ISOOLEFIN/CYCLOPARAFFIN		
37.097	0.12	ISOOLEFIN/CYCLOPARAFFIN		
37.412	0.91	ISOOLEFIN/CYCLOPARAFFIN		
37.482	0.36	NONADECANE	268	C19H40
37.537	0.27	ISOOLEFIN/CYCLOPARAFFIN		
37.837	0.41	ISOOLEFIN/CYCLOPARAFFIN		
37.973	0.44	ISOOLEFIN/CYCLOPARAFFIN		
38.028	0.74	ISOOLEFIN/CYCLOPARAFFIN		
38.263	0.26	ISOOLEFIN/CYCLOPARAFFIN		
38.323	0.35	EICOSANE	282	C20H42
38.573	0.54	ISOOLEFIN/CYCLOPARAFFIN		
39.763	0.17	ISOOLEFIN/CYCLOPARAFFIN		
39.848	0.36	HENEICOSANE	296	C21H44
40.113	0.40	ISOOLEFIN/CYCLOPARAFFIN		
40.479	0.22	ISOOLEFIN/CYCLOPARAFFIN		
40.629	0.65	ISOOLEFIN/CYCLOPARAFFIN		
40.684	0.50	ISOOLEFIN/CYCLOPARAFFIN		
40.989	0.63	ISOOLEFIN/CYCLOPARAFFIN		
41.109	0.52	ISOOLEFIN/CYCLOPARAFFIN		
41.189	0.72	ISOOLEFIN/CYCLOPARAFFIN		
41.239	0.54	ISOOLEFIN/CYCLOPARAFFIN		
41.309	0.62	DOCOSANE	310	C22H46
41.604	0.20	ISOOLEFIN/CYCLOPARAFFIN		
41.694	0.64	ISOOLEFIN/CYCLOPARAFFIN		
42.199	0.12	ISOOLEFIN/CYCLOPARAFFIN		
42.649	0.17	1-DOCOSENE	308	C22H44
42.709	0.39	TRICOSANE	324	C23H48
43.495	0.21	ISOOLEFIN/CYCLOPARAFFIN		
43.58	0.51	ISOOLEFIN/CYCLOPARAFFIN		
43.66	0.18	ISOOLEFIN/CYCLOPARAFFIN		
43.985	0.39	ISOOLEFIN/CYCLOPARAFFIN		
44.065	0.94	ISOOLEFIN/CYCLOPARAFFIN		
44.16	0.23	ISOOLEFIN/CYCLOPARAFFIN		
44.485	0.23	ISOOLEFIN/CYCLOPARAFFIN		
44.56	0.24	ISOOLEFIN/CYCLOPARAFFIN		
45.351	0.21	PENTACOSANE	352	C25H52
45.791	0.15	ISOOLEFIN/CYCLOPARAFFIN		
46.171	0.26	ISOOLEFIN/CYCLOPARAFFIN		
46.261	0.50	ISOOLEFIN/CYCLOPARAFFIN		
46.346	0.24	ISOOLEFIN/CYCLOPARAFFIN		
46.541	0.28	ISOOLEFIN/CYCLOPARAFFIN		
46.611	0.53	HEXACOSANE	366	C26H54

46.731	0.50	ISOOLEFIN/CYCLOPARAFFIN		
47.086	0.21	ISOOLEFIN/CYCLOPARAFFIN		
47.186	0.24	ISOOLEFIN/CYCLOPARAFFIN		
47.812	0.22	HEPTACOSANE	380	C27H56
48.302	0.10	ISOOLEFIN/CYCLOPARAFFIN		
48.642	0.14	ISOOLEFIN/CYCLOPARAFFIN		
48.737	0.30	ISOOLEFIN/CYCLOPARAFFIN		
49.182	0.31	ISOOLEFIN/CYCLOPARAFFIN		
50.112	0.10	NONACOSANE	408	C29H60
51.473	0.14	ISOOLEFIN/CYCLOPARAFFIN		
	4.59	N-PARAFFINS		
	0.17	N-OLEFINS		
	3.77	ISO-PARAFFINS		
	87.06	ISO-OLEFINS		
		CYCLO-PARAFFINS		
	0.17	CYCLO-OLEFINS		
	4.25	AROMATICS		

Table S10. PE/PP/PS/PET/PVC 47.5/47.5/3/1/1 cracking product detailed composition (Sample 10).

RT, min	%wt.	Component name	MW, Da	Structure
3.204	1.78	1-HEPTENE	98	C7H14
3.324	2.31	HEPTANE	100	C7H16
4.839	4.16	TOLUENE	92	C7H8
5.665	1.48	1-OCTENE	112	C8H16
5.99	2.01	OCTANE	114	C8H18
7.56	0.87	CYCLOHEXANE, 1,3,5-TRIMETHYL-, (1.ALPHA, 3.ALPHA,5.BETA)-	126	C9H18
7.66	0.90	2,3-DIMETHYL-2-HEPTENE	126	C9H18
8.036	7.06	2,4-DIMETHYL-1-HEPTENE	126	C9H18
8.991	1.09	CYCLOHEXANE, 1,3,5-TRIMETHYL-	126	C9H18
11.542	0.96	1-NONENE	126	C9H18
11.667	0.86	1-UNDECENE, 8-METHYL-	168	C12H24
12.027	1.69	NONANE	128	C9H20
16.324	1.90	1-DECENE	140	C10H20
16.644	2.02	DECANE	142	C10H22
16.944	1.31	NONANE, 2,6-DIMETHYL-	156	C11H24
17.099	1.46	OCTANE, 2,3,7-TRIMETHYL-	156	C11H24
19.29	2.41	2-DECENE, 2,4-DIMETHYL-	168	C12H24
19.425	1.95	1-DECENE, 2,4-DIMEYTHYL-	168	C12H24
19.735	1.82	1-UNDECENE	154	C11H22
19.985	2.28	UNDECANE	156	C11H24
22.531	1.72	1-DODECENE	168	C12H24
22.736	2.24	DODECANE	170	C12H26
24.992	1.53	1-TRIDECENE	182	C13H26
25.177	2.09	TRIDECANE	184	C13H28
25.237	3.55	1-UNDECENE, 7-METHYL-	168	C12H24
25.432	1.66	ISOOLEFIN/CYCLOPARAFFIN		
25.622	2.89	ISOOLEFIN/CYCLOPARAFFIN		
27.243	1.53	1-TETRADECENE	196	C14H28
27.403	2.05	TETRADECANE	198	C14H30
29.339	1.30	1-PENTADECENE	210	C15H30
29.484	2.19	PENTADECANE	212	C15H32
29.859	1.24	ISOOLEFIN/CYCLOPARAFFIN		
30.049	0.67	ISOOLEFIN/CYCLOPARAFFIN		

30.399	0.76	ISOOLEFIN/CYCLOPARAFFIN		
30.604	0.67	ISOOLEFIN/CYCLOPARAFFIN		
31.31	1.18	CETENE	224	$C_{16}H_{32}$
31.44	2.13	HEXADECANE	226	$C_{16}H_{34}$
33.17	0.97	1-HEPTADECENE	238	$C_{17}H_{34}$
33.296	1.96	HEPTADECANE	240	$C_{17}H_{36}$
33.846	2.30	BENZENE, 1,1'-(2-BUTENE-1,4-DIYL)BIS-	208	$C_{16}H_{16}$
34.356	1.06	ISOOLEFIN/CYCLOPARAFFIN		
34.936	1.36	3-OCTADECENE, (E)-	252	$C_{18}H_{36}$
35.051	1.88	OCTADECANE	254	$C_{18}H_{38}$
36.627	0.96	1-NONADECENE	266	$C_{19}H_{38}$
36.727	1.93	NONADECANE	268	$C_{19}H_{40}$
37.412	1.00	ISOOLEFIN/CYCLOPARAFFIN		
38.232	0.86	3-EICOSENE, (E)-	280	$C_{20}H_{40}$
38.323	1.79	EICOSANE	282	$C_{20}H_{42}$
39.768	0.71	3-HENEICOSENE, (E)-	294	$C_{21}H_{42}$
39.848	1.76	HENEICOSANE	296	$C_{21}H_{44}$
40.628	0.67	ISOOLEFIN/CYCLOPARAFFIN		
41.309	1.65	DOCOSANE	310	$C_{22}H_{46}$
42.709	1.55	TRICOSANE	324	$C_{23}H_{48}$
43.995	0.67	9-TRICOSENE, (Z)-	322	$C_{23}H_{46}$
44.06	1.89	TETRACOSANE	338	$C_{24}H_{50}$
46.601	1.46	HEXACOSANE	366	$C_{26}H_{54}$
47.806	1.26	HEPTACOSANE	380	$C_{27}H_{56}$
48.982	1.12	OCTACOSANE	394	$C_{28}H_{58}$
50.117	0.83	NONACOSANE	408	$C_{29}H_{60}$
51.223	0.67	TRIACONTANE	422	$C_{30}H_{62}$
	40.76	N-PARAFFINS		
	20.72	N-OLEFINS		
	2.77	ISO-PARAFFINS		
	29.28	ISO-OLEFINS		
		CYCLO-PARAFFINS		
	0.00	CYCLO-OLEFINS		
	6.46	AROMATICS		
